# Women with Pregnancies Had Lower Adherence to 1% Tenofovir Vaginal Gel as HIV Preexposure Prophylaxis in CAPRISA 004, a Phase IIB Randomized-Controlled Trial

**DOI:** 10.1371/journal.pone.0056400

**Published:** 2013-03-05

**Authors:** Lynn T. Matthews, Sengeziwe Sibeko, Leila E. Mansoor, Nonhlanhla Yende-Zuma, David R. Bangsberg, Quarraisha Abdool Karim

**Affiliations:** 1 Massachusetts General Hospital, Division of Infectious Disease, Center for Global Health, Boston, Massachusetts, United States of America; 2 Beth Israel Deaconess Medical Center, Division of Infectious Disease, Boston, Massachusetts, United States of America; 3 Harvard Medical School, Boston, Massachusetts, United States of America; 4 Centre for the AIDS Program of Research in South Africa (CAPRISA), Durban, KwaZulu-Natal, South Africa; 5 Mbarara University of Science and Technology, Mbarara, Uganda; 6 Mailman School of Public Health, Columbia University, New York, New York, United States of America; Tulane University, United States of America

## Abstract

**Background:**

Antiretroviral prophylaxis may be a critical strategy to reduce periconception HIV transmission. Maximizing the benefit of periconception pharmacologic HIV risk-reduction requires an understanding of the links between pregnancy and adherence to this prevention strategy.

**Methods:**

We assessed study gel adherence among women with pregnancies compared to women without pregnancies enrolled in the CAPRISA 004 phase IIB trial of 1% vaginal tenofovir gel. Pregnancy was assessed with monthly urine tests. Adherence was measured monthly and defined as proportion of sex acts covered by two returned, used applicators based on pre- and post-coital dosing. High adherence was defined as a median adherence score of >80%, that is, more than 80% of sex acts were covered by two applications of study gel. A multivariate generalized estimating equations (GEE) model with a binomial distribution was used to assess covariates associated with high adherence (>80%) over time. Median adherence before and after pregnancy was compared using Wilcoxon signed rank test.

**Results:**

Among 868 women, 53 had at least 1 pregnancy (4.06 per 100 woman years, 95% CI: 3.04, 5.31). Women with pregnancies had lower median adherence compared to women without pregnancies (50% [IQR: 45–83] vs. 60% [IQR: 50–100], p = 0.02). Women with pregnancies also had a 48% lower odds of high adherence compared to women without pregnancies when adjusting for confounders (aOR 0.52, 95%CI: 0.41–0.66, p<0.0001). Among women with pregnancies, adherence before and after pregnancy was not different (50% [IQR: 46–83] vs. 55% [IQR: 20–100], p = 0.68).

**Conclusions:**

Women with pregnancies were less likely to have high adherence to study gel compared to women without pregnancies. Understanding these differences may inform findings from HIV prevention trials and future implementation of antiretroviral prophylaxis for at-risk women who choose to conceive. The protocol for the parent trial is registered on ClinicalTrials.gov, NCT00441298, http://www.clinicaltrials.gov/ct2/show/NCT00441298.

## Introduction

In sub-Saharan Africa, the majority of new HIV infections occur in women of childbearing age [1]. Antenatal clinic surveys in KwaZulu-Natal, South Africa demonstrate HIV prevalence as high as 46% [2]. While many pregnancies are not planned [3], [4], young women may desire pregnancy to meet her own reproductive goals, cement a relationship with a partner, prove her womanhood and suitability as a future wife, or demonstrate health in an era of HIV-related infertility [5], [6], [7], [8], [9], [10], [11], [12]. Becoming pregnant is risky in settings with high HIV prevalence as current prevention strategies, condoms and abstinence, do not allow for conception. In addition, pregnancy is associated with increased risks of HIV acquisition and transmission [13], [14], [15]. HIV-uninfected women who desire pregnancy with a partner who is HIV-infected or at high risk of being infected require protective strategies for themselves and their future children [16].

Reproductive technologies to reduce periconception HIV transmission risk, including sperm processing, are inaccessible to most [17], [18]. Behavioral strategies such as home manual insemination and unprotected sex limited to peak fertility, and medical strategies including male circumcision [19], [20], [21] and suppressive antiretrovirals (ARVs) for the positive partner [22] are potential elements of periconception HIV risk reduction for serodiscordant couples [23], [24], [25], [26]. Topical or systemic antiretroviral pre-exposure prophylaxis (PrEP) may also be a critical component of strategies to reduce periconception HIV transmission for HIV discordant couples who choose to conceive, particularly if a positive partner does not qualify for or want to take suppressive ARVs [27], [28], [29], [30].

In the first proof of concept microbicide trial, HIV acquisition rates among women using 1% tenofovir vaginal gel were reduced by 39% [31]. HIV acquisition rates were reduced by 44% among HIV-seronegative men or transgender women having sex with men and taking tenofovir/emtricitabine in iPrEX [32]; by 67% and 75% among uninfected partners in HIV-serodiscordant couples taking tenofovir or tenofovir/emtricitabine, respectively, in the Partners PrEP trial [33]; and by 63% among uninfected men and women taking tenofovir/emtricitabine in the CDC TDF2 trial [34]. Conversely, the FEM-PrEP trial of oral daily tenofovir/emtricitabine and the VOICE trial arms of oral and vaginal tenofovir for heterosexual women were halted for futility [35], [36], [37].

Data from these trials suggest that topical or systemic PrEP will work best for those who maintain high medication adherence [31], [32], [33], [35], [38]. Women and couples desiring children may represent a motivated, high-risk population who may benefit from shorter-term PrEP to reduce sexual transmission while allowing for conception [27], [28], [29]. Due to a relative lack of safety data for tenofovir during pregnancy, trials including tenofovir as PrEP in oral or topical formulations have excluded women with plans for pregnancy. Despite extensive counseling and promotion (and, in some cases, provision) of contraceptives, some women in these trials become pregnant [35], [39], [40], [41]. In the Partners PrEP Study of daily oral tenofovir or tenofovir/emtricitabine as PrEP, qualitative interviews with a subset of couples who became pregnant while enrolled suggest a high proportion of intended pregnancies [42]. Examining adherence to ARVs as prevention in PrEP or microbicide trials provides an imperfect but early opportunity to assess potential barriers to periconception pharmacologic risk reduction strategies.

We evaluated pre-pregnancy adherence to study gel among women with pregnancies enrolled in the Centre for the AIDS Programme of Research in South Africa (CAPRISA)- 004 phase IIb placebo-controlled, double-blind randomized clinical trial conducted to assess safety and efficacy of 1% vaginal tenofovir gel to reduce HIV acquisition in women [31]. We hypothesized that women with pregnancies would have higher adherence to study gel compared to women without pregnancies, in the setting of greater perceived risk of HIV acquisition.

## Methods

### Ethics and Regulatory oversight

The CAPRISA 004 trial (NCT00441298) was approved by the University of KwaZulu-Natal's Biomedical Research Ethics Committee (E111/06) and Family Health International's Protection of Human Subjects Committee (#9946). Regulatory oversight was provided by the South African Medicine Control Council (#20060835).

### Study design, population and setting

**Figure 1:**
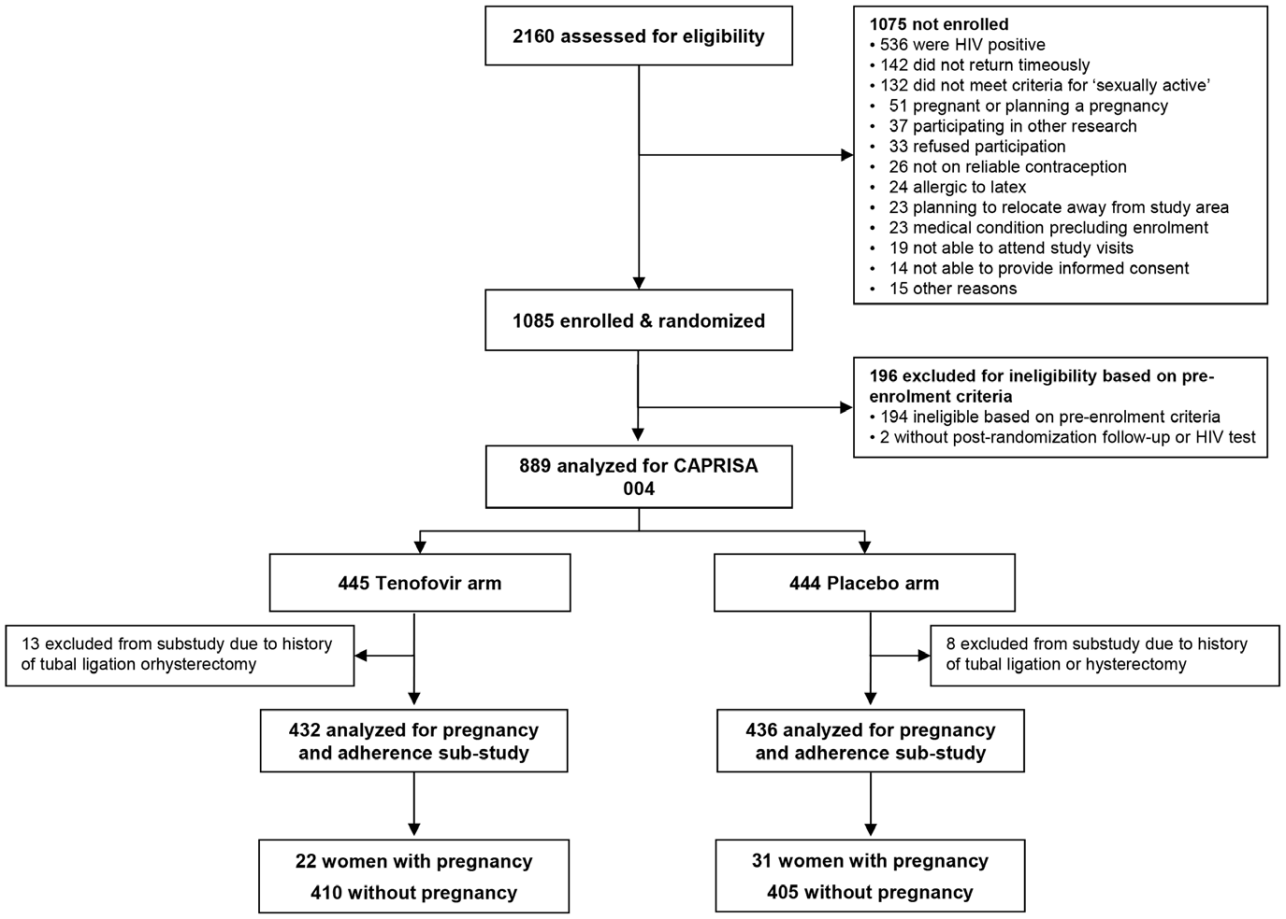
Screening, enrollment, and pregnancy events in the CAPRISA 004 vaginal tenofovir gel trial.

CAPRISA 004 was a phase IIb placebo-controlled, double-blind randomized clinical trial conducted to assess safety and efficacy of 1% vaginal tenofovir gel to reduce HIV acquisition in women at one rural and one urban clinic in South Africa [31]. 889 non-pregnant HIV-uninfected women between the ages of 18 and 40 years were enrolled over 19 months (May 2007 through January 2009) and followed for a mean of 18 months (range: 12 to 30 months). Participants were randomized to receive 1% tenofovir or placebo gel and were instructed to use the gel up to 12 hours before sex, and within 12 hours after sex, without using more than two gels in a 24 hour period. The protocol for this trial is available as supporting information: see Protocol S1. Inclusion criteria included negative response to “Do you have any plans to become pregnant in the next three years?” and agreeing to use a non-barrier method of contraception for the duration of the study. For this analysis, we excluded 21 women who reported a history of hysterectomy or tubal ligation at baseline (Figure 1).

### Pregnancy

Urine pregnancy tests (QuickVue One-Step hCG Urine Test Quidel Corporation, San Diego, USA) were conducted at screening, enrollment (if ≥21 days since screening), and monthly follow-up visits. Pregnant women were excluded from enrollment. Enrolled women who subsequently tested positive for pregnancy continued follow-up visits but study product was temporarily held. Study product resumed following a live birth or once ß-hcg levels in serum or urine reverted to negative.

### Contraception

Hormonal contraceptives were provided on-site at no cost, including progesterone-containing injectables (depot-medroxyprogesterone acetate and norethisterone enanthate) and combined oral contraceptives. Male and female condoms were promoted as an HIV risk-reduction method and were provided at each study visit.

### Adherence

Participants were requested to return all used and unused applicators from the preceding month at each follow-up visit. The number of vaginal sex acts each month was recorded via face-to-face structured interviews. Adherence was calculated by dividing half the number of returned used applicators for each month by the number of reported sex acts for that month. The median of each participant's monthly adherence estimates was assigned as her overall gel adherence score. If a participant reported having no sex in the month, an adherence score was not calculated for that month. High adherence was defined as a median adherence score of >80%, meaning a median of more than 80% of sex acts were covered by two applications of study gel.

### Statistical analysis

In this sub-study, we compared adherence prior to pregnancy among study participants with pregnancies (n = 53) to adherence among study participants without pregnancies (n = 815). Among women with pregnancies who resumed product post-partum (n = 38), we assessed adherence prior to pregnancy and post-partum.

Baseline characteristics of women with and without pregnancies were compared using Fisher's exact test for categorical data and Wilcoxon two-sample test for continuous data. Wilcoxon signed rank test was used to compare median adherence prior to pregnancy and post-partum as well as 0–3 months prior to pregnancy (periconception) and at least 4 months prior to pregnancy (prior to conception). A multivariate generalized estimating equations (GEE) model with a binomial distribution was used to assess covariates associated with high adherence (>80%) over time. Model covariates were selected based on *a priori* knowledge and from unpublished data on predictors of high gel adherence in this trial. Statistical analyses were carried out using SAS (version 9.2; SAS Institute Inc., Cary, NC, USA). All statistical tests were two-sided with type 1 error rate set to 0.05.

## Results

### Baseline characteristics

A total of 868 women were included in this analysis, median age was 22 years (IQR: 20–26), 69% were from the rural study site, 58% had completed high school or above, and 89% reported a stable sexual partner. Participants reported a median of 6 sex acts in the past 30 days (IQR: 4–10), 29% reported always using condoms in past 30 days. Twenty-three percent reported no living children, 54% reported one, and 23% reported at least two living children at enrollment. (Table 1).

**Table 1:** Baseline characteristics of study participants.

Baseline characteristics	All subjects† n = 868	No Pregnancy n = 815	Pregnancy n = 53	p-value*
**Age (years)**, median (IQR)	22 (20–26)	23 (19–27)	22 (20–26)	0.89
**Site**, n (%)				0.45
Rural	597 (68.8)	563 (69.1)	34 (64.2)	
Urban	271 (31.2)	252 (30.9)	19 (35.8)	
**Live with regular partner**, n (%)				0.70
Yes	98 (11.3)	94 (11.5)	4 (7.6)	
No	762 (87.8)	713 (87.5)	49 (92.5)	
No regular partner	8 (0.9)	8 (1.0)	0	
**Education**, n (%)				1.00
Completed high-school and above	506 (58.3)	340 (41.7)	22 (41.5)	
Did not complete high-school	362 (41.7)	475 (58.3)	31 (58.5)	
**1° Partner type**, n (%)				0.34
Married	43 (5.0)	42 (5.2)	1 (1.9)	
Stable ζ	772 (88.9)	721 (88.5)	51 (96.2)	
Other	53 (6.1)	52 (6.4)	1 (1.9)	
**Monthly income**, n (%)				0.44
<145 USD	796 (91.7)	749 (91.9)	47 (88.7)	
≥145 USD	72 (8.3)	66 (8.1)	6 (11.3)	
**Living children**, n (%)				0.26
0	197 (22.7)	180 (22.1)	17 (32.1)	
1	470 (54.1)	445 (54.6)	25 (47.2)	
≥2	201 (23.2)	190 (23.3)	11 (20.8)	
**# Sex acts past 30 days**, median (IQR)	6 (4–10)	6 (4–10)	6 (3–10)	0.69
**Condom use past 30 days**, n (%)				0.53
Always	255 (29.4)	242 (29.7)	13 (24.5)	
Inconsistent	613 (70.6)	573 (70.3)	40 (75.5)	
**Contraception**, n (%)				<0.001
Injectable	730 (84.1)	715 (87.7)	15 (28.3)	
Oral	138 (15.9)	100 (12.3)	38 (71.1)	

† Excluding women reporting prior tubal ligation or hysterectomy.

* Fisher's exact test for categorical data and Wilcoxon two-sample test for continuous data.

ζ Defined to participants as “someone who you have a regular relationship with.”

IQR: interquartile range.

At enrollment 730 women (84%) chose injectable hormonal contraceptives and 138 (16%) chose oral contraceptives. Women on oral contraceptives at baseline were slightly older (24 (IQR: 20–29) vs. 22 (IQR: 20–26) years, p = 0.01), more likely to have completed high school (56% vs. 39% p = 0.001), more likely to have no living children (40% vs. 20%, p<0.0001), and more likely to report monthly household income greater than 1001 South African Rand (∼145 USD) (13% vs. 7%, p = 0.04) compared to women on injectable contraception.

### Pregnancy events

Fifty-three out of 868 women had 54 pregnancies over 30 months (4.06 per 100 woman years of follow-up, 95% CI: 3.04, 5.31) resulting in 35 full-term live births (one set of twins), 4 pre-term deliveries, 10 elective abortions, and 6 miscarriages [41]. Baseline characteristics including age, education, income, number of live births, partner type, condom use, and number of sex acts in past 30 days did not significantly differ between women with and without pregnancies. Women with pregnancies were more likely to be on oral contraception compared to women without pregnancies (71% versus 12%, p<0.0001), as previously reported [41]. (Table 1).

### Association between pregnancy events and adherence to study product

Women with pregnancies (n = 53) were less adherent to study product with median adherence of 50% (IQR 46–83%) compared to women without pregnancies (n = 815) with median adherence of 60% (IQR 50–100%) (p = 0.02). In unadjusted analysis, women with pregnancy had a 42% lower odds of high adherence compared to women without pregnancy (OR 0.58, 95% CI 0.46–0.73, p<.0001). In a multivariate model adjusting for confounders of adherence and pregnancy, women with pregnancy remained less likely to have high adherence to study product (Adjusted OR 0.52, 95%CI: 0.41–0.66; p<0.0001). Contraceptive choice was not associated with adherence to study product (Table 2).

**Table 2:** Covariates associated with adherence greater than 80% (use of 2 applicators with >80% of reported sex acts).

	Odds Ratio (95%CI)	p-value	Adjusted Odds Ratio (95%CI)	p-value
**Ever pregnant (Yes vs No)**	0.58 (0.46–0.73)	<0.0001	0.52 (0.41–0.66)	<0.0001
**Age (per 5 year increase)**	1.06 (1.03–1.10)	0.001	1.15 (1.10–1.20)	<0.0001
**Education (Did not complete vs completed high school)**	1.10 (1.02–1.19)	0.01	0.86 (0.79–0.93)	0.001
**Income (<145 USD vs ≥145 USD)**	2.48 (2.10–2.94)	<0.0001	1.49 (1.21–1.82)	0.0001
**Contraception (Oral vs Injectables)** *	0.96 (0.86–1.06)	0.38	1.06 (0.94–1.19)	0.33
**Site (Rural vs Urban)**	2.57 (2.36–2.80)	<0.0001	2.65 (2.40–2.93)	<0.0001
**Living with regular partner (Yes vs No)**	1.24 (1.11–1.39)	0.0001	1.38 (1.2–1.57)	<0.0001
**Dislike something about gel (Yes vs No)**	0.68 (0.63–0.75)	<0.0001	0.87 (0.79–0.97)	0.01

* Time-updated covariate.

USD  =  U.S. Dollar.

95% CI  = 95% Confidence Interval.

N = 860 for unadjusted comparisons except “dislike something about gel” for which N = 731. N = 731 for the adjusted, multivariate model.

### Adherence before and after pregnancy among those with pregnancy events

Among women with pregnancies who resumed product post-partum (n = 38), median adherence to study drug prior to pregnancy was 50% (IQR 46–83%) compared with 56% (IQR 20–100%) after resumption of study product (p = 0.68). Among women with pregnancies who had adherence data 0–3 months prior to pregnancy (periconception) and ≥4 months prior to pregnancy (n = 30), adherence was 56% (IQR: 50–100%) 0–3 months prior to pregnancy compared with 52% (IQR: 50–83%) ≥4 months prior to pregnancy (p = 0.07).

## Discussion

In this analysis of prospective data from a randomized controlled trial of 1% vaginal tenofovir gel as pre-exposure prophylaxis for HIV prevention in South Africa, women with pregnancies were less adherent to study product than women without pregnancies. When adjusting for available confounders, women with pregnancies had a 48% lower odds of high adherence (two applications of gel with more than 80% of sex acts) to study product prior to pregnancy diagnosis compared to women without pregnancy.

Women with pregnancies may have been less adherent to study product in response to counseling messages. Tenofovir is an FDA class B product [“Adequate, well-controlled studies in pregnant women have not shown an increased risk of fetal abnormalities despite adverse findings in animals, or, in the absence of adequate human studies, animal studies show no fetal risk. The chance of fetal harm is remote, but remains a possibility” [43]] and study participants were counseled about potential risks and taken off study product in the event of pregnancy. This explanation presumes intended pregnancy and informed avoidance of study product. We do not have pregnancy intention data, however, there was no significant difference between adherence prior to pregnancy and post-partum in the subgroup of women with pregnancies, or in periconception (0–3 months prior to pregnancy) adherence compared to timepoints prior to conception (>4 months prior to pregnancy). This may reflect a lack of power to detect differences or suggest that women did not alter gel use in the setting of planning pregnancy.

Women with pregnancies may have been less adherent to both study product and contraception. This may reflect women who enrolled in the study for secondary benefits but without particular interest in the study product, or women who were simply not able to adhere to study product or contraception. The only noted difference between women with and without pregnancies at enrollment was contraceptive choice. We do not have contraception adherence data but women on oral contraception – which requires daily adherence – were more likely to become pregnant than women on injectables. Interestingly, women on oral contraceptives were slightly older and more likely to have completed high school, have no children, and report monthly household income greater than 145 USD than women on injectable contraception. These women may have been prepared to have children and may have chosen a contraceptive method which they could readily control. If choice of oral contraception marked pregnancy desire, this choice did not impact adherence to study gel in the model, again suggesting that women did not avoid study product in the setting of planning pregnancy. In addition, while some women switched contraceptive methods during the study, only two women with pregnancies switched from injectables to oral contraceptives prior to her pregnancy. Of note, among the three women with pregnancies who acquired HIV, all were in the placebo arm [41]. Additional details regarding contraceptive use, HIV seroconversion, and pregnancy have been published [41]. A manuscript on the predictors of adherence (besides pregnancy) in this study is in preparation.

These data address two points for future prevention trials. The rate of pregnancy in this study (4.06 per 100 woman years of follow-up, 95% CI: 3.04, 5.31) is lower than that observed in other HIV prevention trials [39], [40], [44], [45], [46], [47], [48] but illustrates that some women who do not plan pregnancy and have excellent access to contraception become pregnant. Prevention studies must anticipate pregnancies and incorporate analyses to understand the nature of the pregnancy (e.g. intention, partner role in decision), adherence to study drug, and pregnancy outcomes in order to understand efficacy and safety. In this study, choice of or switch to oral contraception was associated with pregnancy. This choice may have indicated desire for pregnancy or simply increased the risk for pregnancy given reliance on daily pill taking. Studies that depend on low pregnancy rates to preserve power to assess product efficacy may consider requiring longer-acting contraception, but this is complicated by observed associations between injectable hormonal contraception and HIV risk [49], [50], [51], [52], [53], [54]. Given that antiretroviral PrEP is one of few prevention options for uninfected women who want to conceive a child with an infected or high-risk partner [27], [28], [29], [30], including women with pregnancy or plans for pregnancy in prevention studies may ultimately be more efficient than working to exclude them. Second, our data suggest that women with pregnancies were less likely to maintain high adherence to study product. Prospective data to understand adherence in this context are needed.

There are limitations to these data. First, our ability to explain differences in adherence among women with pregnancies versus those without is compromised by an inability to accurately assess pregnancy intention or desire. Inclusion criteria for this study included a negative answer to the question “Do you plan to become pregnant in the next three years?” However, while most women in this study were probably not planning to become pregnant at enrollment, pregnancy planning is not static and many pregnancies fall between the extremes of explicitly planned and unplanned [12]. Second, studying adherence to an intervention with unknown efficacy with blinded randomization to placebo is challenging and makes both the measure of adherence and the significance of the finding challenging to interpret. In addition, adherence data for women with pregnancy events were censored at pregnancy. Thus, women with pregnancies had a median of 7.9 (IQR: 5.0–15.4) months of follow-up and women without pregnancies had a median of 19.2 (IQR: 14.6–23.2) months of follow-up. We would expect this differential follow-up time to bias our results in the opposite direction of our findings (higher adherence among women with pregnancies) given that medication adherence tends to decay over time [55], [56]. This study was not designed to answer questions of acceptability or efficacy of periconception PrEP use. These preliminary findings however provide some important considerations for future studies.

## Conclusions

Antiretroviral prophylaxis may be an important component of interventions to reduce periconception HIV transmission. In this study, women with pregnancies were less adherent to study product. This may reflect unintended pregnancy in the context of imperfect contraceptive adherence and associated poor study gel adherence. Concerns about tenofovir toxicity may have also led to product nonuse for women who decided to conceive and wanted to reduce fetal exposure. Further research is needed to understand the relationship between antiretroviral prophylaxis adherence, pregnancy intention, and concerns about fetal toxicity. In addition, efforts to develop adherence support strategies for PrEP and to assess feasibility of periconception PrEP use for at-risk women who choose to conceive remain crucial.

## Supporting Information

Protocol S1 (PDF)
